# Associations of Mediterranean Diet Adherence, Dietary Glycemic Index, and Antioxidant Intake With Acne Vulgaris: A Case–Control Study in Young Adults

**DOI:** 10.1111/jocd.71256

**Published:** 2026-10-28

**Authors:** Ayçıl Özturan‐Şirin, Aydan Yazıcı

**Affiliations:** ^1^ Department of Nutrition and Dietetics, Faculty of Health Sciences Aydın Adnan Menderes University Aydın Turkey; ^2^ Department of Dermatology, Faculty of Medicine Aydın Adnan Menderes University Aydın Turkey

**Keywords:** acne vulgaris, dietary antioxidant score, dietary patterns, glycemic index, MEDAS, Mediterranean diet, young adults

## Abstract

**Background:**

Dietary patterns, glycemic characteristics, and oxidative balance have been implicated in acne vulgaris, although findings remain inconsistent.

**Aim:**

This study evaluated the associations of Mediterranean diet adherence, dietary glycemic index (GI), and dietary antioxidant score with acne vulgaris in young adults.

**Methods:**

This case–control study included 279 participants (115 acne/164 controls). Dietary intake was assessed using three‐day food records, and Mediterranean diet adherence was evaluated with the Mediterranean Diet Adherence Screener (MEDAS). Individual MEDAS components were compared using Holm adjustment. Two forced‐entry multivariable logistic regression models assessed associations with acne status while adjusting for age, BMI, sex, education, and vitamin/mineral supplementation.

**Results:**

Participants with acne had lower MEDAS scores than controls (5.97 ± 2.22 vs. 7.02 ± 2.12; *p* < 0.001), whereas dietary GI did not differ between groups (57.57 ± 4.99 vs. 57.74 ± 7.13; *p* = 0.821). Dietary antioxidant scores were higher in the acne group (3.74 ± 2.53 vs. 3.02 ± 2.46; *p* = 0.019). After Holm correction, only the vegetable and butter/cream/margarine criteria remained significantly different. Failure to meet these criteria was independently associated with higher odds of acne (aOR = 3.242 and 2.413, respectively). Higher MEDAS scores were inversely associated with acne (aOR = 0.806; 95% CI: 0.710–0.915), while higher dietary antioxidant scores showed a modest positive association (aOR = 1.126; 95% CI: 1.008–1.258). Dietary GI was not independently associated with acne (aOR = 1.049; 95% CI: 0.685–1.607).

**Conclusions:**

Lower Mediterranean diet adherence was associated with higher odds of acne vulgaris, whereas dietary GI was not independently associated. The positive association observed for dietary antioxidant score should be interpreted cautiously given the case–control design and potential residual confounding.

## 1 Introduction

Acne vulgaris is a chronic inflammatory skin disease with a complex and multifactorial pathogenesis involving increased sebum production, androgen‐mediated hormonal activity, follicular hyperkeratinization, and bacterial proliferation. It is among the most common dermatologic disorders worldwide, with an estimated global prevalence of approximately 9.4% [1, 2]. Acne predominantly affects adolescents and young adults, and studies have reported that approximately 80%–100% of individuals between 11 and 30 years of age experience acne during their lifetime [3, 4, 5]. However, acne is no longer considered exclusively a disease of adolescence, as increasing prevalence has also been reported among adults [6, 7].

Although acne primarily manifests as a dermatological condition, its impact extends beyond physical appearance. Visible lesions and residual scarring may negatively affect psychological well‐being and quality of life by contributing to anxiety, depressive symptoms, social isolation, and impaired self‐esteem [8]. Due to both its high prevalence and psychosocial burden, acne vulgaris represents an important public health concern [9].

In recent years, increasing attention has been directed toward the relationship between dietary factors and acne. Several studies have suggested an association between Western dietary patterns and acne prevalence [10, 11]. Diets characterized by high consumption of refined carbohydrates, dairy products, saturated fats, and ultra‐processed foods have been proposed to influence pathways involved in acne development. Changes in dietary and lifestyle patterns have been proposed as potential contributors to the increasing burden of acne [10].

Among dietary factors, glycemic characteristics have been investigated extensively because of their potential biological role in acne pathogenesis. High‐glycemic diets may increase insulin and insulin‐like growth factor‐1 (IGF‐1) signaling, potentially activating the mammalian target of rapamycin complex 1 (mTORC1) pathway and promoting sebum production and keratinocyte proliferation [10]. In addition, interleukin‐mediated inflammatory responses play an important role in acne pathogenesis [12]. Consistent with these proposed mechanisms, several studies have demonstrated positive associations between dietary glycemic factors and acne occurrence or severity [13, 14, 15, 16]. Similarly, a systematic review by Meixiong et al. [11] reported that most observational studies identified associations between glycemic index (GI), glycemic load, and acne‐related outcomes.

In contrast to Western dietary patterns, the Mediterranean diet is characterized by high consumption of vegetables, fruits, legumes, whole grains, olive oil, nuts, and seafood, while emphasizing lower intake of red meat and highly processed foods [17, 18]. Because of its anti‐inflammatory and antioxidant properties, adherence to the Mediterranean diet has been proposed as a potentially protective factor against acne vulgaris [19]. The Mediterranean diet has been associated with anti‐inflammatory effects, which may be relevant to the inflammatory processes involved in acne pathogenesis [20]. Separately, experimental evidence has demonstrated the involvement of IGF‐1‐related signaling pathways in keratinocyte proliferation, sebaceous activity, and inflammatory responses [21, 22, 23]. Furthermore, a recent meta‐analysis reported a significant inverse association between Mediterranean diet adherence and acne severity [19].

Therefore, this study aimed to evaluate the associations of Mediterranean diet adherence, dietary GI, dietary antioxidant score, and selected dietary components with acne vulgaris among young adults.

## 2 Materials and Methods

### 2.1 Study Design and Participants

This face‐to‐face, questionnaire‐based case–control study was conducted among young adults to investigate the associations of Mediterranean diet adherence, dietary GI, dietary antioxidant intake, and selected dietary components with acne vulgaris. The study was reported in accordance with the Strengthening the Reporting of Observational Studies in Epidemiology (STROBE) guidelines.

Patients diagnosed with acne vulgaris were recruited from individuals attending the Dermatology Outpatient Clinic of Aydın Adnan Menderes University Hospital during the study period. The control group consisted of individuals without a diagnosis of acne vulgaris who attended the same outpatient clinic during the corresponding study period. Following completion of participant recruitment, the final study population comprised 279 individuals, including 115 patients with acne vulgaris and 164 controls.

To improve comparability between the study groups, recruitment was extended to obtain a more balanced age distribution. In the final sample, age did not differ significantly between patients with acne vulgaris and controls. Because educational attainment remained different between the groups, education was retained as a potential confounding variable in all multivariable models.

Participation was voluntary. Individuals were eligible if they were young adults, did not have a psychiatric disorder, and were not following a disease‐specific or otherwise prescribed dietary regimen. Pregnant or lactating individuals, those following a special dietary program, and individuals unwilling to participate were excluded.

This study protocol was approved by the Ethics Committee of the Faculty of Health Sciences, Aydın Adnan Menderes University, under protocol number 2025/36 and numbered E‐15189967‐050.04‐731 962. Written informed consent was obtained from all participants before enrollment. This study adhered to the ethical principles outlined in the Declaration of Helsinki and relevant national and institutional ethical regulations.

### 2.2 Data Collection

Data were collected through face‐to‐face interviews using a structured questionnaire comprising six sections: sociodemographic characteristics, general health information, dietary habits, Mediterranean diet adherence, acne‐related quality of life, and dietary intake records.

Information on age, sex, educational attainment, marital status, chronic disease status, medication use, vitamin/mineral supplementation, smoking, alcohol consumption, and meal‐related habits was obtained by self‐report. Height and body weight were also self‐reported rather than measured directly. Body mass index (BMI) was calculated as body weight in kilograms divided by height in meters squared (kg/m^2^).

BMI was evaluated both as a continuous variable and descriptively according to standard BMI categories. For multivariable regression analyses, BMI was entered as a continuous variable to avoid loss of information and unstable estimates arising from small numbers in individual BMI categories.

### 2.3 Assessment of Mediterranean Diet Adherence

Mediterranean diet adherence was assessed using the Mediterranean Diet Adherence Screener (MEDAS), originally developed by Martínez‐González et al. and subsequently validated in Turkish by Pehlivanoğlu et al. [18, 24]. Each of the 14 MEDAS items was scored as 0 or 1 according to whether the corresponding dietary criterion was met, yielding a total score ranging from 0 to 14. Higher scores indicated greater adherence to the Mediterranean diet.

According to the Turkish validation study, participants with a total MEDAS score < 7 were classified as non‐adherent to the Mediterranean diet, whereas those with scores ≥ 7 were classified as adherent. The total MEDAS score was additionally analyzed as a continuous variable in the multivariable regression models. Individual MEDAS components were also evaluated. For negatively framed food components, including red/processed meat, butter/cream/margarine, and sweet/carbonated beverages, a score of 1 indicated that the recommended lower‐intake criterion was met.

### 2.4 Assessment of Acne‐Related Quality of Life

Acne‐related quality of life was assessed using the Acne Quality of Life Scale developed by Gupta et al. and validated in Turkish by Demirçay et al. [25, 26]. The scale consists of nine items with a total score ranging from 9 to 36, where higher scores indicate greater impairment in quality of life.

### 2.5 Assessment of Acne Severity

Acne severity was assessed using the Global Acne Grading System (GAGS). The GAGS evaluates acne severity by considering six anatomical locations, including the forehead, right cheek, left cheek, nose, chin, and chest/upper back. Each region is assigned a factor according to its surface area and lesion distribution. Lesion types, including comedones, papules, pustules, and nodules, are graded according to severity, and a total score is calculated by summing regional scores. Based on total scores, acne severity was categorized as mild (1–18), moderate (19–30), severe (31–38), or very severe (> 38) [27].

For secondary analyses, moderate and severe cases were combined because of the limited number of participants in the higher‐severity categories. Accordingly, patients were classified as having mild or moderate‐to‐severe acne. All acne severity assessments were performed by the same dermatologist, who was blinded to participants' dietary assessment data. Analyses according to acne severity were considered exploratory because of the relatively limited subgroup sample sizes.

### 2.6 Dietary Assessment

Dietary intake was assessed using 3‐day food records comprising two weekdays and one weekend day. Participants recorded foods and beverages consumed during these 3 days using an individual food consumption record form. Dietary records were subsequently analyzed using the Nutrition Information System (BeBiS) software.

Dietary GI and dietary antioxidant scores were derived from the dietary records. For the primary analyses, the average dietary values obtained from the 3‐day records were used.

Dietary GI values were assigned to carbohydrate‐containing foods primarily using the International Tables of Glycemic Index and Glycemic Load Values 2021 by Atkinson et al. [28]. When an exact GI value was unavailable, a structured food‐matching approach adapted from Aston et al. [29] was applied, whereby values were assigned using a comparable food or, when necessary, an appropriate food‐group estimate. For selected traditional Turkish foods, glucose‐referenced GI values reported in a Hacettepe University master's thesis were used, including simit (60.0), bazlama (70.2), and whole‐wheat bread (51.3) [30]. Thus, the assigned GI values included directly matched reference values as well as comparable‐food and food‐group estimates.

For each participant, dietary GI was estimated using a carbohydrate‐weighted approach. The GI value assigned to each food item was multiplied by the amount of carbohydrate contributed by that food, and the sum of these products was divided by total carbohydrate intake.

When no suitable direct, comparable‐food, or food‐group match could be identified for a carbohydrate‐containing food, the food was not assigned a GI value of zero. Instead, a nominal GI value of 70 was assigned to the remaining unmatched carbohydrate intake, consistent with the assignment approach described by Aston et al. [29]. This nominal value represents an analytical assumption rather than a directly measured GI value. Accordingly, the resulting dietary GI values should be interpreted as source‐based estimates derived from dietary records rather than direct measurements of the glycemic responses to the meals consumed by the participants.

Dietary GI was analyzed as a continuous variable. To improve clinical interpretability of the regression estimates, dietary GI was scaled in 10‐unit increments in multivariable analyses.

### 2.7 Statistical Analysis

Statistical analyses were performed using IBM SPSS Statistics version 22.0 (IBM Corp., Armonk, NY, USA). Continuous variables were assessed for distributional characteristics and are presented as mean ± standard deviation, whereas categorical variables are presented as number and percentage.

Between‐group comparisons of continuous variables were performed using independent‐samples *t*‐tests, whereas categorical variables were compared using Pearson's chi‐squared test. Fisher's exact test was used when expected cell counts were insufficient for the chi‐squared approximation.

Because the 14 individual MEDAS components were evaluated simultaneously, Holm's sequential adjustment procedure was applied to account for multiple comparisons. Both crude and Holm‐adjusted *p* values are reported for these comparisons. Statistical significance for the individual MEDAS component comparisons was interpreted on the basis of the Holm‐adjusted *p* values.

Two separate multivariable binary logistic regression models were constructed to evaluate factors associated with acne status. Acne status was coded as 1 for acne vulgaris and 0 for controls. To avoid data‐driven stepwise variable selection, all variables specified for each model were entered simultaneously using the forced‐entry (Enter) method.

The first model examined selected MEDAS dietary components and included age, BMI, sex, educational attainment, vitamin/mineral supplementation, vegetable intake criterion, butter/cream/margarine criterion, and sweet/carbonated beverage criterion. BMI was modeled as a continuous variable to reduce the number of model parameters and avoid sparse BMI category cells.

The second model evaluated overall dietary measures and included age, BMI, sex, educational attainment, vitamin/mineral supplementation, MEDAS total score, dietary antioxidant score, and dietary GI. MEDAS was expressed as the change in odds associated with a 1‐point increase in the total score. For greater interpretability of the effect estimate, dietary GI was rescaled and modeled per 10‐unit increase.

Adjusted odds ratios (aORs) with corresponding 95% confidence intervals (95% CIs) are reported. A two‐sided *p* value < 0.05 was considered statistically significant, except for the individual MEDAS component comparisons, for which Holm‐adjusted *p* values were used.

## 3 Results

### 3.1 Participant Characteristics

A total of 279 participants were included in the final analysis, comprising 164 controls and 115 individuals with acne vulgaris. The mean age was 23.98 ± 6.74 years in the control group and 22.70 ± 5.08 years in the acne group, with no statistically significant between‐group difference (*p* = 0.087). Sex distribution, marital status, chronic disease status, medication use, BMI category, and mean BMI were also comparable between groups (all *p* > 0.05). However, educational attainment differed significantly between groups; a greater proportion of participants with acne had a high school education or below compared with controls (30.4% vs. 13.4%, *p* = 0.001). Vitamin/mineral supplementation was also less frequent in the acne group than in the control group (12.2% vs. 25.0%, *p* = 0.005) (Table 1).

**TABLE 1:** Sociodemographic, clinical, and dietary characteristics of patients with acne vulgaris and controls.

Variable	Control (*n* = 164)	Acne vulgaris (*n* = 115)	*p*
Age (*x̄* ± ss)	23.98 ± 6.74	22.70 ± 5.08	0.087
Sex, *n* (%)			
Male	21 (12.8)	15 (13.0)	0.953
Female	143 (87.2)	100 (87)	
Marital status, *n* (%)			
Single	145 (88.4)	108 (93.9)	0.120
Married	19 (11.6)	7 (6.1)	
Education, *n* (%)			
High school or below	22 (13.4)	35 (30.4)	0.001
College/university or above	142 (86.6)	80 (69.6)	
Chronic disease, *n* (%)			
Yes	28 (17.1)	27 (23.5)	0.186
No	136 (82.9)	88 (76.5)	
Medication use, *n* (%)			
Yes	25 (15.2)	23 (20.0)	0.300
No	139 (84.8)	92 (80)	
Vitamin/mineral supplementation, *n* (%)			
Yes	41 (25.0)	14 (12.2)	0.008
No	123 (75.0)	101 (87.8)	
BMI category, *n* (%)			
Underweight	28 (17.1)	12 (10.4)	0.428
Normal	98 (59.8)	75 (65.2)	
Overweight	29 (17.7)	23 (20.0)	
Obese	9 (5.5)	5 (4.4)	
Mediterranean diet adherence, *n* (%)			
Non‐adherent (< 7)	70 (42.7)	77 (67.0)	< 0.001
Adherent (≥ 7)	94 (57.3)	38 (33.0)	
BMI, kg/m^2^ (*x̄* ± ss)	22.25 ± 3.95	22.82 ± 4.36	0.264
MEDAS total score (*x̄* ± ss)	7.02 ± 2.12	5.97 ± 2.22	< 0.001
Dietary glycemic index (*x̄* ± ss)	57.74 ± 7.13	57.57 ± 4.99	0.821
Dietary antioxidant score (mmol/day) (*x̄* ± ss)	3.02 ± 2.46	3.74 ± 2.53	0.019

*Note:* Data are presented as mean ± standard deviation or *n* (%). *p*‐values for continuous variables were obtained using independent‐samples *t*‐tests, and categorical variables were compared using Pearson's chi‐squared test. Statistical significance was defined as *p* < 0.05.

Abbreviations: BMI, body mass index; MEDAS, Mediterranean Diet Adherence Screener.

Mediterranean diet adherence differed significantly between groups. Non‐adherence to the Mediterranean diet (MEDAS < 7) was observed in 67.0% of participants with acne compared with 42.7% of controls (*p* < 0.001). Consistently, the mean MEDAS total score was significantly lower in the acne group than in the control group (5.97 ± 2.22 vs. 7.02 ± 2.12, *p* < 0.001). In contrast, dietary GI did not differ between the groups (57.57 ± 4.99 vs. 57.74 ± 7.13, *p* = 0.821). The dietary antioxidant score was higher among participants with acne than among controls (3.74 ± 2.53 vs. 3.02 ± 2.46, *p* = 0.019) (Table 1).

### 3.2 Comparison of MEDAS Components Between Groups

Analysis of individual MEDAS components showed several crude between‐group differences; however, after Holm correction for multiple comparisons, only two components remained statistically significant. Meeting the criterion of consuming at least two servings of vegetables per day was less common among participants with acne than among controls (33.0% vs. 64.6%, Holm‐adjusted *p* < 0.001). Similarly, meeting the criterion of consuming less than one serving per day of butter, cream, or margarine was less frequent in the acne group (40.9% vs. 64.0%, Holm‐adjusted *p* = 0.002). Although the sweet or carbonated beverage criterion differed in the crude analysis (*p* = 0.005), this association did not remain statistically significant after Holm correction (adjusted *p* = 0.057). The tree nut criterion was also significant only in the crude analysis (*p* = 0.043; Holm‐adjusted *p* = 0.469) (Table 2).

**TABLE 2:** Comparison of Mediterranean Diet Adherence Screener (MEDAS) components between patients with acne vulgaris and controls.

MEDAS component (criterion met)	Control (*n* = 164), *n* (%)	Acne vulgaris (*n* = 115), *n* (%)	Crude *p*	Holm‐adjusted *p*
Use of olive oil as main source of fat	125 (76.2)	87 (75.7)	0.913	1.000
Olive oil ≥ 4 tablespoons/day	97 (59.1)	57 (49.6)	0.113	1.000
Vegetables ≥ 2 servings/day	**106 (64.6)**	**38 (33.0)**	**< 0.001**	**< 0.001**
Fruits ≥ 3 servings/day	41 (25.0)	34 (29.6)	0.397	1.000
Red or processed meats < 1 serving/day	81 (49.4)	51 (44.3)	0.406	1.000
Butter, cream, or margarine < 1 serving/day	**105 (64.0)**	**47 (40.9)**	**< 0.001**	**0.002**
Sweet or carbonated beverages < 1 cup/day	88 (53.7)	42 (36.5)	0.005	0.057
Legumes ≥ 3 servings/week	6 (3.7)	2 (1.7)	0.477*	1.000
Wine consumption ≥ 7 glasses/week	88 (53.7)	51 (44.3)	0.126	1.000
Fish and seafood ≥ 3 servings/week	19 (11.6)	9 (7.8)	0.304	1.000
Commercial sweets ≤ 2/week	101 (61.6)	70 (60.9)	0.904	1.000
Tree nuts ≥ 3 servings/week	71 (43.3)	36 (31.3)	0.043	0.469
Poultry more than red meats	99 (60.4)	73 (63.5)	0.599	1.000
Use of sofrito sauce in a dish ≥ 2 servings/week	125 (76.2)	89 (77.4)	0.820	1.000

*Note:* Data are presented as *n* (%). Between‐group comparisons were performed using Pearson's chi‐squared test, except where expected cell counts were < 5, for which Fisher's exact test was used. *p*‐values were adjusted for multiple comparisons across the 14 MEDAS components using the Holm procedure.

Abbreviation: MEDAS, Mediterranean Diet Adherence Screener. The values in bold indicate statistically significant results.

* Fisher's exact test.

### 3.3 Association of Selected MEDAS Components With Acne Vulgaris

In the multivariable logistic regression model examining selected MEDAS components, educational attainment and two dietary components were independently associated with acne status. Participants with a high school education or below had higher odds of being in the acne group compared with those with college/university education or above (aOR = 2.504, 95% CI: 1.269–4.939, *p* = 0.008). Not meeting the vegetable intake criterion was associated with higher odds of acne (aOR = 3.242, 95% CI: 1.882–5.585, *p* < 0.001), as was not meeting the butter/cream/margarine criterion (aOR = 2.413, 95% CI: 1.397–4.167, *p* = 0.002). The sweet or carbonated beverage criterion was not independently associated with acne status (aOR = 1.496, 95% CI: 0.861–2.598, *p* = 0.153). Age, BMI, sex, and vitamin/mineral supplementation were also not statistically significant in this model. The model was significant overall (*χ* ^2^(8) = 58.792, *p* < 0.001), with a Nagelkerke *R* ^2^ of 0.256 and an overall classification accuracy of 72.0% (Table 3).

**TABLE 3:** Multivariable logistic regression analysis of selected dietary components associated with acne vulgaris.

Variable	Comparison/unit	aOR	95% CI	*p*
Age	Per 1‐year increase	0.955	0.907–1.005	0.077
BMI	Per 1 kg/m^2^ increase	1.030	0.959–1.107	0.415
Sex	Male vs. female	0.861	0.384–1.930	0.716
Education	High school or below vs. college/university or above	2.504	1.269–4.939	0.008
Vitamin/mineral supplementation	Yes vs. no	0.553	0.268–1.141	0.109
Vegetable intake criterion	Not met vs. met	3.242	1.882–5.585	< 0.001
Butter/cream/margarine criterion	Not met vs. met	2.413	1.397–4.167	0.002
Sweet/carbonated beverage criterion	Not met vs. met	1.496	0.861–2.598	0.153

*Note:* Outcome variable was acne status (1 = acne vulgaris, 0 = control). All variables were entered simultaneously using the forced‐entry method.

Abbreviations: aOR, adjusted odds ratio; CI, confidence interval; BMI, body mass index; MEDAS, Mediterranean Diet Adherence Screener.

### 3.4 Associations of Mediterranean Diet Adherence, Dietary Glycemic Index, and Dietary Antioxidant Score With Acne Vulgaris

In the second multivariable logistic regression model, lower educational attainment remained associated with higher odds of acne (aOR = 2.835, 95% CI: 1.471–5.466, *p* = 0.002). Higher MEDAS scores were inversely associated with acne status (aOR = 0.806 per 1‐point increase, 95% CI: 0.710–0.915, *p* = 0.001). Expressed in the opposite direction, each 1‐point decrease in MEDAS score corresponded to approximately 24.1% higher odds of acne. The dietary antioxidant score was positively associated with acne status (aOR = 1.126 per 1‐unit increase, 95% CI: 1.008–1.258, *p* = 0.035). In contrast, dietary GI was not associated with acne status when expressed per 10‐unit increase (aOR = 1.049, 95% CI: 0.685–1.607, *p* = 0.826). Age, BMI, sex, and vitamin/mineral supplementation were not statistically significant. The model was significant overall (*χ* ^2^(8) = 40.517, *p* < 0.001), with a Nagelkerke *R* ^2^ of 0.182 and an overall classification accuracy of 65.2% (Table 4).

**TABLE 4:** Multivariable logistic regression analysis of Mediterranean diet adherence, dietary glycemic index, and dietary antioxidant score associated with acne vulgaris.

Variable	Comparison/unit	aOR	95% CI	*p*
Age	Per 1‐year increase	0.955	0.908–1.003	0.066
BMI	Per 1 kg/m^2^ increase	1.038	0.968–1.112	0.292
Sex	Male vs. female	0.851	0.382–1.896	0.693
Education	High school or below vs. college/university or above	2.835	1.471–5.466	0.002
Vitamin/mineral supplementation	Yes vs. no	0.526	0.255–1.083	0.081
MEDAS total score	Per 1‐point increase	0.806	0.710–0.915	0.001
Dietary antioxidant score	Per 1‐unit increase	1.126	1.008–1.258	0.035
Dietary glycemic index	Per 10‐unit increase	1.049	0.685–1.607	0.826

*Note:* Outcome variable was acne status (1 = acne vulgaris, 0 = control). All variables were entered simultaneously using the forced‐entry method. For interpretability, MEDAS is presented per 1‐point increase and dietary glycemic index per 10‐unit increase.

Abbreviations: aOR, adjusted odds ratio; CI, confidence interval; BMI, body mass index; MEDAS, Mediterranean Diet Adherence Screener.

### 3.5 MEDAS Components According to Acne Severity

In the exploratory analysis restricted to participants with acne vulgaris, none of the individual MEDAS components differed significantly between those with mild acne (*n* = 37) and those with moderate‐to‐severe acne (*n* = 78) after correction for multiple comparisons (Table S1).

## 4 Discussion

In the present case–control study, lower adherence to the Mediterranean diet was associated with acne vulgaris in young adults. Participants with acne vulgaris had lower MEDAS scores than controls, and this association remained significant after adjustment for age, BMI, sex, educational attainment, vitamin/mineral supplementation, dietary antioxidant score, and dietary GI. In the fully adjusted model, each 1‐point decrease in the MEDAS total score was associated with approximately 24.1% higher odds of acne vulgaris. In addition, among the individual MEDAS components, failure to meet the vegetable intake criterion and failure to meet the butter/cream/margarine criterion were independently associated with higher odds of acne vulgaris. By contrast, dietary GI was not independently associated with acne status. Dietary antioxidant score was higher among participants with acne and showed a modest positive association with acne status after multivariable adjustment. These findings should be interpreted as associations rather than causal effects because of the observational case–control design.

The inverse association observed between Mediterranean diet adherence and acne vulgaris is broadly consistent with previous studies suggesting a potential relationship between Mediterranean dietary patterns and acne‐related outcomes. The Mediterranean diet is characterized by relatively high consumption of vegetables, fruits, legumes, whole grains, olive oil, nuts, and seafood, together with lower consumption of red meat and highly processed foods [17, 18]. Its anti‐inflammatory and antioxidant characteristics have been proposed to influence pathways potentially relevant to acne pathogenesis [19, 20, 21, 22, 23]. Barrea et al. [31] reported lower Mediterranean diet adherence among patients with acne and identified an association between lower PREDIMED scores and greater acne severity. Similarly, Ah‐Thiane et al. [32] reported an inverse relationship between Mediterranean diet adherence and facial acne severity in French women. A recent systematic review and meta‐analysis also suggested an inverse association between Mediterranean diet adherence and acne severity [19]. Nevertheless, the literature is not entirely consistent. Taha et al. [33] found no significant relationship between MEDAS scores and acne diagnosis or severity among university students, whereas Aryanian et al. [34] reported associations primarily with selected Mediterranean diet components rather than a consistent relationship with acne occurrence. Differences in study populations, dietary cultures, dietary assessment methods, and acne‐related characteristics may partly explain these heterogeneous findings. In the present study, MEDAS was evaluated primarily as a continuous score in the multivariable analysis, which may provide a more informative assessment than reliance on categorical adherence thresholds alone.

In contrast to some previous evidence, dietary GI was not associated with acne vulgaris in the present study. Mean dietary GI values were similar between participants with acne and controls, and no independent association was observed after adjustment for age, BMI, sex, educational attainment, vitamin/mineral supplementation, Mediterranean diet adherence, and dietary antioxidant score (aOR = 1.049 per 10‐unit increase, 95% CI: 0.685–1.607, *p* = 0.826). Previous studies have nevertheless reported associations between dietary glycemic characteristics and acne‐related outcomes. Çerman et al. [14] reported significantly higher dietary GI and glycemic load values among individuals with acne vulgaris than among controls, while the systematic review by Meixiong et al. [11] concluded that most observational studies examining GI or glycemic load reported associations with acne occurrence or severity. Furthermore, Smith et al. [16] demonstrated improvements in acne lesions among participants assigned to a low‐glycemic‐load dietary intervention, providing experimental support for a potential role of dietary glycemic characteristics in acne. Diet‐related factors in acne have similarly emphasized high‐glycemic dietary patterns as a potentially relevant dietary characteristic [35].

High‐glycemic dietary patterns may contribute to metabolic signaling pathways implicated in acne through postprandial hyperinsulinemia, increased IGF‐1 signaling, and downstream pathways related to sebaceous lipogenesis and keratinocyte proliferation [10]. However, the present findings did not demonstrate an association between dietary GI and acne status. Differences from previous studies may reflect variation in study populations, dietary patterns, dietary assessment methods, or the distribution of GI values. In particular, the relatively similar mean GI values observed between the groups in the present study may have limited the ability to detect an association. In addition, measurement error inherent in short‐term self‐reported dietary records cannot be excluded. Thus, although previous biological and epidemiological evidence provides a rationale for investigating glycemic dietary characteristics in acne, the present findings do not support an independent association between dietary GI and acne status in this sample.

Among the individual MEDAS components, vegetable intake showed one of the most robust associations with acne status. The proportion of participants meeting the MEDAS vegetable criterion (≥ 2 servings/day) was significantly lower in the acne vulgaris group than in the control group, and this difference remained significant after Holm correction for multiple comparisons. In the multivariable model, participants who did not meet the vegetable intake criterion had more than threefold higher odds of acne vulgaris than those who met the criterion (aOR = 3.242, 95% CI: 1.882–5.585). Previous studies examining Mediterranean dietary patterns and acne have also suggested that greater consumption of plant‐based foods, including vegetables, may be associated with more favorable acne‐related outcomes [31, 32, 34]. In addition, reviews of dietary factors in acne have emphasized the potential relevance of diets rich in vegetables, fruits, vitamins, and antioxidant compounds [35].

Vegetables provide dietary fiber, vitamins, carotenoids, polyphenols, and other compounds with antioxidant and anti‐inflammatory properties. Given the involvement of oxidative stress and inflammation in acne pathophysiology, these dietary characteristics provide biological plausibility for the observed association [35, 36]. Zujko‐Kowalska et al. [36] also reported favorable quality‐of‐life findings among young women with acne consuming diets with higher antioxidant capacity. Nevertheless, the present findings do not establish a protective effect of vegetable consumption. Vegetable intake may also be a marker of a broader dietary and lifestyle pattern, and residual confounding by other behavioral or nutritional characteristics cannot be excluded.

Although the dietary antioxidant score was significantly higher in the acne vulgaris group in the unadjusted comparison, it also remained positively associated with acne status after multivariable adjustment (aOR = 1.126, 95% CI: 1.008–1.258, *p* = 0.035). This finding was unexpected and should not be interpreted as evidence that higher dietary antioxidant intake increases the risk of acne. Previous findings on antioxidant‐related dietary measures and acne have also been inconsistent. Zujko‐Kowalska et al. [36] reported that greater dietary antioxidant capacity was associated with more favorable quality‐of‐life outcomes among young women with acne, whereas Majdizadeh et al. [37] did not identify a significant association between dietary oxygen radical absorption capacity and acne‐related outcomes in adolescents. These heterogeneous findings may reflect differences in study populations, antioxidant assessment methods, and the specific outcomes evaluated.

The positive association observed for dietary antioxidant score in the present study does not contradict a potential role of oxidative stress or antioxidant‐rich foods in acne pathophysiology, nor does it establish a detrimental effect of antioxidant intake. The dietary antioxidant score may capture multiple aspects of food intake and may be influenced by broader dietary patterns, total food consumption, or other unmeasured characteristics. Reverse causality is also possible, as individuals with acne may modify their food choices after disease onset. Accordingly, this finding should be interpreted cautiously and requires confirmation in prospective studies. More broadly, antioxidant exposure may be more appropriately considered within the context of the overall dietary pattern rather than as an isolated dietary score [35, 36, 37].

No statistically significant differences were detected in any of the individual MEDAS components between participants with mild acne and those with moderate‐to‐severe acne. Previous studies examining Mediterranean diet adherence in relation to acne severity have yielded inconsistent findings. Barrea et al. [31] reported an inverse association between Mediterranean diet adherence and acne severity, and Ah‐Thiane et al. [32] similarly observed an inverse relationship between Mediterranean diet adherence and facial acne severity. In contrast, Taha et al. [33] found no significant association between MEDAS scores and acne severity among university students. More recently, Aryanian et al. [34] reported that Mediterranean diet adherence was significantly associated with acne severity among patients, although overall adherence scores did not significantly differ between acne cases and controls. These inconsistencies may reflect differences in study populations, dietary assessment methods, and approaches to acne severity classification. Moreover, acne severity represents a multifactorial clinical outcome that may coexist with psychological symptoms and altered eating behaviors, further complicating the interpretation of diet–severity relationships [38].

Importantly, the absence of statistically significant differences in the present study should not be interpreted as evidence of the absence of an association between Mediterranean diet components and acne severity. The severity analysis included only patients with acne vulgaris and was based on relatively small subgroups (37 participants with mild acne and 78 with moderate‐to‐severe acne), which limited the statistical power to detect modest associations. Accordingly, these analyses were considered exploratory, and larger studies with adequate statistical power across acne severity categories are needed to clarify whether Mediterranean diet adherence or its individual components are associated with acne severity.

The findings of the present study should be interpreted in light of the case–control design, which does not allow the temporal direction of the observed associations to be established. Reverse causality is therefore possible. Individuals with acne may modify their dietary habits after disease onset because of personal beliefs, medical advice, or exposure to information regarding diet and acne. In addition, the psychosocial burden of acne, including impaired quality of life, anxiety, social withdrawal, and other psychological symptoms, may influence eating behavior and food choices [8, 9, 38]. Consequently, the dietary characteristics observed among participants with acne may, at least in part, reflect behavioral changes occurring after acne development rather than dietary exposures that preceded disease onset.

Although educational attainment differed between the groups and was independently associated with acne status, the principal Mediterranean diet‐related associations remained significant after adjustment for education and age. This suggests that the observed associations with Mediterranean diet adherence and the vegetable and butter/cream/margarine criteria were not solely attributable to these between‐group differences. Residual confounding should also be considered. Although the multivariable models adjusted for age, BMI, sex, educational attainment, and vitamin/mineral supplementation, several potentially relevant factors were not comprehensively assessed in the present study. These include hormonal characteristics, menstrual cycle‐related factors, oral contraceptive use, psychological stress, sleep quality, physical activity, and detailed histories of topical or systemic acne treatment. Because some of these factors may be associated with both dietary behavior and acne‐related outcomes, their absence from the analytic models may have contributed to residual confounding. Therefore, the adjusted associations reported in the present study should not be interpreted as fully independent of all potential confounders.

The association between diet and acne vulgaris has been investigated previously, particularly in relation to glycemic characteristics, Western dietary patterns, and Mediterranean diet adherence [11, 19, 31, 32, 33, 34, 35]. Therefore, the contribution of the present study does not lie in proposing a previously unrecognized relationship between diet and acne. Rather, this study adds evidence from a Turkish young‐adult population by evaluating Mediterranean diet adherence, individual MEDAS components, dietary GI, and dietary antioxidant score within the same analytical framework.

An additional contribution of the present analysis is the simultaneous evaluation of both overall dietary pattern measures and individual MEDAS components. The revised analyses also incorporated adjustment for multiple comparisons across the 14 MEDAS items and used prespecified forced‐entry multivariable models rather than data‐driven stepwise variable selection. In this context, the findings may help clarify which dietary characteristics show the most consistent associations with acne status within this population, while remaining complementary to the existing literature rather than constituting evidence of a novel causal relationship.

The present study has several strengths. Acne diagnosis and severity were evaluated clinically, and all GAGS assessments were performed by the same dermatologist independently of the participants' dietary information, reducing the potential for differential severity assessment according to dietary exposure. Dietary characteristics were evaluated using both an overall Mediterranean diet adherence score and individual MEDAS components, together with dietary GI and dietary antioxidant score. In addition, the revised statistical approach accounted for multiple comparisons across individual MEDAS components and used prespecified forced‐entry multivariable models, thereby reducing reliance on data‐driven variable selection. The MEDAS total score and dietary GI were also evaluated as continuous measures in the primary multivariable analysis, avoiding exclusive reliance on categorical dietary classifications.

Several limitations should be considered when interpreting these findings. First, the case–control design precludes conclusions regarding temporality or causality and does not exclude reverse causality, as individuals with acne may modify their dietary habits after disease onset. Second, dietary assessment was based on self‐reported 3‐day food records, which may be affected by reporting error and may not fully represent habitual intake; objective dietary or metabolic biomarkers were not available. The use of comparable‐food values, food‐group estimates, and a nominal GI value for unmatched foods may have introduced uncertainty into the dietary GI estimates. Height and weight were also self‐reported, introducing potential BMI misclassification. Although the multivariable analyses accounted for age, BMI, sex, educational status, and vitamin/mineral supplementation, residual confounding by unmeasured factors, including hormonal and menstrual factors, oral contraceptive use, stress, sleep, physical activity, and acne treatment history, cannot be excluded. In addition, the butter/cream/margarine MEDAS item represents a composite criterion and therefore does not allow the independent effects of these foods to be distinguished. The acne severity subgroup analysis should be regarded as exploratory because of its limited sample size. Finally, the single‐center design and inclusion of predominantly young Turkish adults may limit the generalizability of the findings to populations with different demographic, cultural, and dietary characteristics.

Taken together, the present findings support an association between overall dietary patterns and acne vulgaris rather than the influence of a single dietary factor. Lower Mediterranean diet adherence remained associated with acne status after adjustment for the measured covariates, while vegetable intake and the composite butter/cream/margarine criterion emerged as the individual MEDAS components showing the most robust associations. Dietary GI was not associated with acne status, whereas dietary antioxidant score showed a modest positive association after multivariable adjustment. No statistically significant differences in individual MEDAS components were detected according to acne severity. Given the potential for reverse causality, residual confounding, and dietary measurement error, these findings should be interpreted as observational associations. Prospective studies with repeated dietary assessments and more comprehensive measurement of potential confounders, as well as appropriately designed dietary intervention studies, are needed to clarify the temporal and potentially causal nature of these relationships.

Lower Mediterranean diet adherence was independently associated with higher odds of acne vulgaris in this case–control study. Among individual Mediterranean diet components, not meeting the vegetable intake criterion and not meeting the criterion for limited butter, cream, or margarine consumption were also associated with higher odds of acne vulgaris. Dietary GI was not independently associated with acne status, whereas a modest positive association was observed for dietary antioxidant score.

These findings suggest that overall dietary characteristics may be associated with acne status; however, the observational design precludes conclusions regarding temporality or causality. Therefore, the results should not be interpreted as evidence that modification of these dietary factors prevents or treats acne vulgaris. Prospective cohort studies and controlled dietary intervention trials are needed to determine whether the observed associations have causal or clinical relevance.

## Author Contributions

A.Ö.‐Ş.: conceptualization, methodology, formal and statistical analysis, writing – original draft, writing – review and editing. A.Y.: conceptualization, data curation, methodology, formal analysis, writing – review and editing. The authors have approved the final version submitted.

## Funding

The authors have nothing to report.

## Ethics Statement

This study was approved by the Ethics Committee of the Faculty of Health Sciences, Adnan Menderes University, under protocol number 2025/36 and numbered E‐15189967‐050.04‐731 962.

## Consent

Informed consent was obtained from all individual participants included in the study. The participants (parents/guardians) have agreed for their anonymized data/information to be published.

## Conflicts of Interest

The authors declare no conflicts of interest.

## Supporting information


**Table S1:** Comparison of MEDAS components according to acne severity among patients with acne vulgaris.

## Data Availability

The data that support the findings of this study are available on request from the corresponding author. The data are not publicly available due to privacy or ethical restrictions.
