# Supplementary material for: Associations of Mediterranean Diet Adherence, Dietary Glycemic Index, and Antioxidant Intake With Acne Vulgaris: A Case–Control Study in Young Adults
Source: J Cosmet Dermatol. 2026 Oct 28;25(11):e71256. doi: 10.1111/jocd.71256 (PMC13715055; doi:10.1111/jocd.71256)
Supplement: Supplementary file 1 [file JOCD-25-e71256-s001.docx]

**Table S1.** Comparison of MEDAS Components According to Acne Severity Among Patients With Acne Vulgaris

| **MEDAS component** | **Mild (n=37), n (%)** | **Moderate-to-severe (n=78), n (%)** | **Crude P-value** | **Holm-adjusted P-value** |
| --- | --- | --- | --- | --- |
| Use of olive oil as the main source of fat | 24 (64.9) | 63 (80.8) | 0.063 | 0.882 |
| Olive oil ≥4 tablespoons/day | 19 (51.4) | 38 (48.7) | 0.792 | 1.000 |
| Vegetables ≥2 servings/day | 9 (24.3) | 29 (37.2) | 0.171 | 1.000 |
| Fruits ≥3 servings/day | 9 (24.3) | 25 (32.1) | 0.396 | 1.000 |
| Red/processed meat criterion | 21 (56.8) | 30 (38.5) | 0.065 | 0.882 |
| Butter, cream, or margarine criterion | 15 (40.5) | 32 (41.0) | 0.961 | 1.000 |
| Sweet/carbonated beverage criterion | 12 (32.4) | 30 (38.5) | 0.530 | 1.000 |
| Legumes criterion | 0 (0.0) | 2 (2.6) | 1.000* | 1.000 |
| Wine criterion | 16 (43.2) | 35 (44.9) | 0.870 | 1.000 |
| Fish/seafood criterion | 2 (5.4) | 7 (9.0) | 0.716* | 1.000 |
| Commercial sweets/pastries criterion | 21 (56.8) | 49 (62.8) | 0.534 | 1.000 |
| Tree nuts criterion | 12 (32.4) | 24 (30.8) | 0.857 | 1.000 |
| Poultry consumed more frequently than red meat | 24 (64.9) | 49 (62.8) | 0.832 | 1.000 |
| Sofrito criterion | 26 (70.3) | 63 (80.8) | 0.209 | 1.000 |

**Note:** Data are presented as n (%). Comparisons were performed using Pearson's chi-square test or Fisher's exact test where expected cell counts were small. *Fisher's exact test. MEDAS, Mediterranean Diet Adherence Screener.
